# The Search for Predictive Biomarkers in Response to Immune Checkpoint Inhibitors and Associated Adverse Events

**DOI:** 10.3390/jpm15120596

**Published:** 2025-12-03

**Authors:** Marco Agostini, Pietro Traldi, Mahmoud Hamdan

**Affiliations:** Corso Stati Uniti 4, Istituto di Ricerca Pediatrica Città della Speranza, 35100 Padova, Italy

**Keywords:** biomarkers, immune checkpoint inhibitors, immune-related adverse events, mass spectrometry-based proteomics

## Abstract

The introduction of immune checkpoint inhibitors (ICIs) as a part of immunotherapy represented a therapeutic breakthrough in the landscape of cancer treatment. The action of these inhibitors consists of blocking certain inhibitory receptors in the immune system. Blocking these inhibitory pathways, ICIs induce an enhanced T cell-mediated response necessary to neutralize tumor cells. Over the last 10 years, programmed death cell protein1 (PD-1), PD ligand 1 (PD-L1), and cytotoxic T lymphocyte-associated antigen 4 (CTLA-4) have been among the inhibitory receptors most targeted by ICIs. Currently, this innovative therapeutic approach faces two major challenges: early identification of cancer patients who are likely to get a significant therapeutic benefit through the use of these inhibitors, and the second challenge is the early prediction of likely immune-related adverse events (irAEs) associated with such therapy. The aim of the present text is to discuss the current research efforts to discover and develop much needed effective biomarkers, which may represent an important step towards more efficient and risk-free immunotherapy. We also highlight the increasing role in clinical analyses of liquid biopsy sampling combined with mass spectrometry-based proteomics and how such combination is contributing to current research efforts to enhance the role of immunotherapy.

## 1. Introduction

The introduction of immune checkpoint inhibitors (ICIs) as part of immunotherapy, over 10 years ago, is still considered a major therapeutic leap in the treatment of a number of malignant tumors. The therapeutic action of these inhibitors has been mainly tested against few of a long list of known checkpoints: Cytotoxic T lymphocyte-associated antigen 4 (CTLA-4), programmed death cell protein (PD-1), PD ligand 1 (PD-L1), and lymphocyte activation gene 3 (LAG-3). By blocking certain inhibitory pathways associated with these checkpoints, ICIs induce an enhanced immune response towards invading tumor cells. Numerous clinical trials together with other sources of clinical data have shown that ICIs face two main challenges: (a) the discovery and development of effective biomarkers for an accurate stratification of patients who can get significant benefit from the therapy; and (b) the discovery and development of biomarkers for an early and accurate prediction of likely immune-related adverse events (irAEs) associated with ICIs therapy. It is now well recognized that the use of ICIs can provoke a wide spectrum of toxicities (see Table 1).

These adverse events can manifest with different degrees of severity at any time during the treatment and even after its termination [9,10,11]. irAEs are generally graded using a common terminology. In the case of grade 1 events, the ICIs treatment is not interrupted, while grade 2 or higher necessitate the cessation of the treatment and the start of corticosteroids or other immunomodulators [12,13]. The search for predictive biomarkers to anticipate the onset of these adverse events is ongoing. The discovery and validation of such biomarkers would facilitate a more personalized approach and more effective patient management. Over the last few years, there have been different approaches to identify such biomarkers. These approaches included serum antibody profiling prior to ICIs treatment using a human microarray [14,15] and serial monitoring of Cytokine expression in plasma samples of patients treated with ICIs [16]. These approaches are treated in more details in the discussion.

The identification of reliable predictive biomarkers for ICIs treatment of anti-PD-1/PD-L1 and CTL-4 remains one of the priorities of immunotherapy. It is therefore imperative to identify biomarkers that discriminate between responders and non-responders to ICIs therapy. Currently, there are three biomarkers, which have been approved by the FDA as predictive biomarkers: PD-L1 expression on tumor cells, microsatellite instability/defective mismatch repair (MSI/dMMR), and tumor mutational burden (TMB), indicated in Table 2 [17,18,19]. Clinical trials investigating the efficacy of these predictive biomarkers have demonstrated that none of these biomarkers on their own can provide accurate assessment of patient response.

Such discouraging clinical results accelerated the search for more effective biomarkers. It is worth bearing in mind that the identification of these biomarkers has been based on investigations limited to anti-PD-1/PD-L1 and CTLA-4. Current research efforts involve a long list of emerging checkpoints (see Table 3).

**Figure 1:**
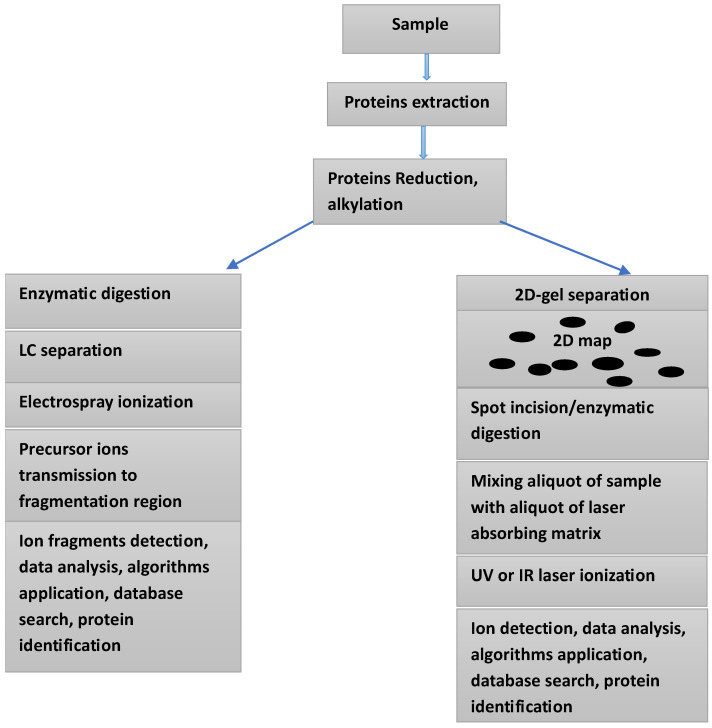
Two approaches for the analysis of a protein’s mixture within a given sample: Electrospray ionization combined with LC-MS/MS (**left side**), and 2D-gel electrophoresis combined with MALDI-TOF mass spectrometry (**right side**).

Mass spectrometry-based proteomics is considered an indispensable tool in the search for biomarkers for various serious disease, including different forms of cancer and neurodegenerative and cardiovascular diseases. Using this technique to investigate body fluids and other biological samples can furnish crucial information on protein quantification, identification, and structural characterization, including post-translational modifications (PTMs) and protein/protein and protein/ligand interactions. Given that the main players in ICIs therapy are mainly proteins, the use of mass spectrometry-based proteomics is rendered an important tool in the process of biomarkers discovery and validation. The availability of high resolution, high mass accuracy mass spectrometers, and a substantial progress in sample preparation have contributed to an increased use of this technique, particularly in clinical settings. Furthermore, the increasing use of liquid biopsy (LB) sampling has enforced the use of MS analysis of biofluids. Various investigations, including numerous clinical trials have indicated that the search for biomarkers for both irAEs and ICIs therapy requires serial sampling followed by precise and fast analyses, two characteristics of the combination LB sampling and MS-based proteomics. Currently, mass spectrometry (MS) combined with liquid chromatography (LC) is the main analytical platform for the analysis of complex protein mixtures within various biological samples (see Figure 1). Entering into a detailed discussion of the recent development in mass spectrometry and its applications in the medical field is outside the scope of this review. The recent literature offers many excellent works on the topic [20,21,22,23,24].

### The Emerging Role of Spatial Multi-Omics

For many years single-cell sequencing has been instrumental in providing valuable and detailed insight into gene expression at the individual cell level. The high resolution of this approach allows detailed characterization of cell diversity. However, this powerful method is incapable of providing information on the spatial contest among cell components. This limitation is gradually addressed by the increasing use of spatial multi-omics technologies. More recently, spatial omics have emerged as a powerful method for predicting treatment responses and monitoring tumor evolution. These technologies enable a deeper understanding of cellular organization and various interactions within diseased and healthy tissues. The last decade has witnessed a substantial increase in the development and commercialization of spatially resolved omics technologies. Most such technologies allow the visualization and quantitation of numerous molecular targets and cellular components within tissue while maintaining the tissue architecture intact. This detailed spatial information is fundamental for deeper understanding of tumor heterogeneity and of the tumor microenvironment; both parameters are pivotal for a better understanding of tumor resistance, response to therapy, and the discovery of more specific predictive biomarkers for irAEs associated with new therapeutic regimes. More details on these emerging technologies have been given in a number of recent works, and we see no need to repeat such details here [25,26,27]. That said, the application of these powerful technologies for analyses relevant to immune therapy, including the discovery and development of a new generation of predictive biomarkers, is likely to give much needed support to this area of therapy. The same technologies will certainly give additional momentum to the identification of more promising therapeutic targets, leading to a new class of immune therapies.

## 2. Discussion

### 2.1. Observations Regarding Researched Immune Checkpoints (Inhibitory Immunoreceptors)

Unlike conventional cancer therapies such as chemotherapy and radiotherapy, immune therapy works by enforcing the immune system, allowing it to fight more efficiently when invading tumor cells. Table 3 lists a few of the immune checkpoints discovered and studied in cancer over the last two decades. These molecules function as gate keepers of the immune responses, which explains the use of the term “immune checkpoints” [28]. Targeting these molecules with monoclonal antibodies is considered one of the success stories in cancer therapies [29]. These molecules have an important regulatory role over the responses of the immune system. Once present in cancer cells, these molecules would facilitate cancer cells’ evasion of the immune system. Some immune cell inhibitors have demonstrated to be an efficient therapeutic method to interrupt such evasion. Over the last 10 years, the inhibition of immune checkpoints, such as cytotoxic T lymphocyte antigen-4, programmed cell death-1, and programmed cell death ligand-1, has taken a leading role in immune therapy. This relatively recent therapy regime is based on the use of checkpoint inhibitors, which enhance the immune response towards various forms of cancer. The prevailing opinion regarding immune therapy suggests that for this approach of therapy to deliver on its promise, a number of challenges have to be circumvented. Among these is included the need to understand the resistance mechanisms to immune checkpoint blockade. Such understanding requires accurate identification of the various signaling pathways which impact the functions and the role of these checkpoints within the immune system. For example, the Phosphatidylinositol 3-kinase (PI3K)/Akt pathway is one of the critical signaling pathways targeted by PD-1. This pathway is known to participate in many cellular processes, such as proliferation and apoptosis, and it is the main target of the suppressive function of PD-1 [29,30,31,32]. More details on the relevance of this and other signaling pathways have been obtained in a number of works dealing with the role of different checkpoints in immune therapy [29,30,31,32].

### 2.2. Some Approved Predictive Biomarkers

Clinical data generated over the last 15 years have demonstrated that ICIs treatment is only effective in less than 30% of patients with advanced stages of certain forms of cancer [33,34,35,36]. The majority of these patients either did not respond or developed resistance to these inhibitors. The high cost of these inhibitors and the serious irAEs associated with them underline the urgent need for predictive biomarkers capable of accurate identification of patients who are likely to get a significant therapeutic benefit using these inhibitors. Currently, there are three predictive biomarkers which have been approved by the FDA to predict response to ICIs, two of these biomarkers are considered below:

#### 2.2.1. Cell Death Ligand-L1 (PD-L1)

The expression level of PD-L1 in tumor tissues or in liquid biopsies of various cancer patients is considered the main predictive biomarker in response to anti-PD-1/PD-L1 therapy. The predictive efficacy of this biomarker, based on its level of expression, still raises a strong debate fueled by contradictory results regarding such efficacy. A number of studies have shown that the expression of PD-L1 in some NSCLC patients was low or even negative, yet the response to anti-PD-1/PD-L1 was better than the response of other patients in which high expression of the same biomarker was observed [37,38,39]. Numerous investigations, including various clinical trials, demonstrated that PD-L1 immunohistochemistry for measuring PDL-1 expression still represents a challenging task, as it requires an understanding of a complex issue encompassing multiple clones, tested by multiple platforms for multiple different malignancies, each with variable scoring criteria and different thresholds [40,41]. The approval by the FDA of four different assays to assess such expression did not contribute to attempts to standardize such measurements. Such necessary standardization is still difficult to implement when the approved methods use different PD-L1 expression thresholds, different scoring systems, different antibodies, and different cells expressing this biomarker. Another confounding factor which impacts the accuracy of PD-L1 expression is the heterogeneity of such expression when measured on tissue samples, an effect which can be attenuated by using liquid biopsy rather than tissue sampling. The question of assay standardization, particularly at the clinical level, is bound to reduce or even eliminate inconstant measurements.

#### 2.2.2. Tumor Mutational Burden (TMB)

TMB was approved by the FDA in 2020 as a predictive biomarker in response to ICIs treatment of unresectable or metastatic solid tumors. This biomarker refers to the total number of somatic non-synonymous mutations present within a cancer genome. Both retrospective and prospective studies have supported the use of TMB as a predictive biomarker in response to ICIs. As in the case of PDL-1, this biomarker still demonstrates inconsistent results in its clinical applicability across different types of cancer.

Such uncertainty has been fueled by inconsistency between the TMB measurements generated by different platforms and different assays [42,43,44]. The use of the TMB level as a predictive biomarker and the approval of pembrolizumab monotherapy by the FDA for the subgroup of solid tumor patients were mainly based on the results of the “KEYNOTE-158” trial [45]. This trial reported that patients with previously treated, unresectable or metastatic solid tumors, having TMB-high status (<10 mutations per mega base), showed a clinically meaningful improvement in the efficacy of pembrolizumab against PD-1 checkpoints [46]. In a recent study, TMB measurements were performed for over 8000 patients, suffering from 24 cancer types [47]. This was the largest study to examine the association between TMB and real-world overall survival (rwOS) of a number of participants. This study concluded that patients with TMB ≥ 10 mut/Mb level, receiving anti-PD-L1 monotherapy have shown favorable rwOS across different tumor types compared to patients with lower levels of TMB. In an earlier study, a cohort of about 700 patients with 8 distinct advanced cancer diagnoses examined the correlation between TMB level and overall survival [48,49]. This study concluded that TMB-high cancers were significantly associated with longer overall survival than TMB-low cancers. Based on the present-day literature, TMB as a predictive biomarker is limited by a number of elements: (i) The predictive threshold of TMB is still the center of a strong debate. Based on a solid tumor subgroup treated with ICIs, the Kenote-158 trial suggested <10 mutations per mega base as the predictive level, while other investigations reported ≥20 TMB level as the only predictive threshold for patients receiving a combination of ICI-chemotherapy [50,51,52]. The variation in TMB level, reported by different studies, is not surprising. An analysis of 100,000 human cancer genomes revealed an interesting TMB landscape [53,54]. The authors reported that below 0.5 Mb the variation in measured TMB increased significantly, and a subset of patients exhibited high TMB across almost all types of cancer. The same study showed that TMB increased significantly with age, where a 2.4-fold difference was observed in the age between 10 and 90 years. (ii) Both prospective and retrospective studies have shown mixed clinical results and failed to demonstrate consistent overall survival benefit [55,56]. TMB can be accurately assessed by using whole-genome sequencing (WGS); however, this method is very costly, has a relatively long time of implementation, and requires sufficient tissue; these characteristics have interfered with its clinical diffusion [57,58,59]. Instead, multiple commercially available gene panels designed for TMB estimation are commonplace in clinical practice. It is well known that commercial panel-based sequencing of tumor tissue has different sizes, different mutation types, and different bioinformatic tools. These differences have to be carefully considered and evaluated before reaching conclusions regarding the consistency of TMB levels generated by different clinical trials in which different panel-based sequencings were used.

The two predictive biomarkers discussed above have been in clinical use for a number of years, yet both have a number of limitations which interfere in their performance as effective biomarkers in the prediction of ICIs response. It is fair to say that these limitations are mainly associated with the diversity of the assays of detection, absence of standardization, and the insistence of using a single biomarker to address problems in which multiple markers are necessary. Currently, there is not sufficient convincing clinical data to suggest that the combination of these two biomarkers can result in significant improvement in the predictability of the response to ICIs treatment. Based on existing yet limited data, such improvement is not to be expected. PD-L1 expression and TMB are not significantly correlated within most cancer subtypes, and they show only a marginal association at the tumor level. Furthermore, the two biomarkers have no overlapping effects on the response rate to PD-1/PD-L1 inhibitors [60,61]. In a recent investigation, the predictive value of PD-L1 and TMB for short-term efficacy prognosis in non-small cell lung cancer (NSCLC) was assessed. The data of patients receiving first-line treatment with anti-PD-1 immune checkpoint inhibitors combined with chemotherapy were retrospectively collected [61]. Multivariate analyses in this study showed that PD-L1, TMB, and neutrophil were independent prognostic indicators for the objective response rate group (ORR). The areas under the receiver operating characteristic (ROC) for the three indicators were used to construct a prediction model and, according to the authors, a model based on the three readings gave better predictions than models based on a single indicator. These conclusions need to be supported by further studies, involving higher number of patients and possibly other forms of cancer.

### 2.3. The Search for New Predictive Biomarkers

Limitations of the three predictive biomarkers approved by the FDA and existing gaps in therapeutic strategies, which mainly target four checkpoints, have underlined the urgent need to explore the potential of other proteins (described as emerging checkpoints), not only as new predictive biomarkers but also as candidates for new therapeutic strategies (see Table 2). Interest in some of these emerging checkpoints is based on their overexpression in various tumor tissues and their limited expression in normal tissues. Two representative examples of these emerging checkpoints are considered below.

#### 2.3.1. B7 Homolog 3 Protein (B7-H3, Also Known as CD276)

B7-H3 is glycoprotein, which belongs to the B7 family. The basic structure of this protein contains a pair of extracellular immunoglobulin variable-like (IgV) and immunoglobulin constant-like (IgC) domains (IgV-IgC), a transmembrane region, and a short cytoplasmic tail containing 45 amino acids with a signal peptide at its amino (-NH2) terminus. Human B7-H3, exists in two isoforms, which have the molecular weights of 50 (2IgB7-H3) and 100 k Da (4IgB7-H3), respectively [62]. This protein is considered one of the emerging immune checkpoints and is known to participate in different forms of upregulation in solid tumors and in various hematologic diseases [63]. Furthermore, there is accumulating evidence to suggest that this protein contributes to immune evasion through its suppression of natural killer (NK) and T cell functions. Other roles attributed to this protein include promotion of cancer cell migration, invasion, metastasis, and resistance to chemotherapy [64,65,66]. These indicated functions rendered it an attractive target for both therapeutic strategies and as a prognostic biomarker for a number of solid tumors [67,68,69]. To underline this statement, it is worth considering some of the works, which investigated some of these functions. In one of these investigations the authors compared the proteomes of two subpopulations of glioblastoma cells, where the differential expression of various proteins was assessed. The authors used different methods of analysis, including Western blotting (WB), quantitative real-time polymerase chain reaction (qRT-PCR), Immunostaining, and public mRNA expression databases for gene expression analysis [70]. This study concluded that the lighter isoform of B7-H3, 21 g B7-3H, was associated with the more aggressive phenotype of glioblastoma cells. The same authors hypothesized that such association may enhance the recurrence of the disease. This hypothesis was mainly based on higher level of 2IgB7-3H expression recurrences compared to newly diagnosed disease. This hypothesis merits the following considerations: differentiation between the two isoforms is important for the accuracy of the assessment, particularly if such investigations are used for patient stratification and for decision making regarding a specific therapy. That said, Immunostaining of tissues to determine the level of expression of the target isoform can be influenced by the heterogeneity of such expression on tissues. This effect was clearly observed in the case of the PD-L1 analysis. It was observed that the heterogeneity of PD-L1 expression within a given tumor or between different tumors can negatively impact the accuracy of the measurement. Conducting such measurements in liquid biopsy samples rather than on tissues was found to attenuate such negative effect [71,72,73].

In another study, the sandwich enzyme-linked immunosorbent assay (ELISA) was used to measure B7-3H levels in the serum of colon cancer patients [74]. Protein levels were measured in preoperative serum obtained from 90 patients and compared with the levels measured in 50 controls. This investigation concluded that B7-3H levels were significantly higher in colon cancer patients compared to controls. The same study reported that the level of expression of this protein was significantly higher in patients in stage IV of the disease compared to patients in stages I and II. These conclusions merit a number of observations: First, these results have been generated using a very limited number of participants, with no follow up. Second, human B7-H3 is known to have two isoforms, 2Ig-B7-H3 and 4Ig-B7-H3. The same literature indicates that 4Ig-B7-H3 is the dominant isoform in the human body and is linked to immune evasion mechanisms in the tumor microenvironment. Furthermore, there is no substantial evidence to suggest that the lighter isoform, 2Ig-B7-H3, is involved in tumor immunity in humans [75,76]. The absence of any reference to these isoforms in the work in this study renders reported results less informative. It is well recognized that protein PTMs play crucial roles in many biochemical and biological events, such as protein folding, degradation, cellular localization, intracellular transport, and protein–protein interactions. The impact of B7H3 glycosylation on its mediation of various functions was clearly demonstrated in recent studies [77,78,79]. These studies have demonstrated that various amino acids within the sequence of B7H3 experience PTMs. These modifications involve asparagine (N), lysine (K), serine (S), and threonine (T). The most frequent and most investigated among these modifications is glycosylation at eight different sites: N91, N104, N189, N215, N309, N322, N407, and N433. In addition to Glycosylation, two other modifications were reported, phosphorylation at S513 and T551 along with ubiquitination at the sites K521 and K526 [79]. The impact of these glycosylated asparagines on the function of B7H3 are still not fully understood; however, in a recent study it was demonstrated that glycosylated asparagine residues, N91/309 and N104/322 are required for proper B7H3 localization on the cell surface membrane. The same group provided evidence that the glycosylation of N91/309 and N104/322 is essential for B7H3 to inhibit T cell proliferation and activation. Based on these results, the same group developed the glycosylation-specific B7H3 antibody, Ab-82, which according to this group preferentially targets N-glycosylated B7H3 at N91/309 and N104/322, and demonstrate that Ab-82 exerts favorable effects on antitumor immunity by inducing B7H3 degradation [80]. In their study the authors used a long list of analytical techniques including quantitative real-time PCR, flow cytometry, immunoblotting and immunoprecipitation, immunohistochemical staining, liquid chromatography-mass spectrometry (LC-MS), and immunofluorescence. In an earlier study, the same research group investigated the potential role of glycosylated B73H within a strategy to enhance immune checkpoints therapy. The authors investigated such potential role in the treatment of triple-negative breast cancer (TNBC). This form of cancer is a heterogeneous subtype of breast cancer that generally has a poor prognosis, with high rates of systemic recurrence or metastatic potential and refractoriness to conventional therapy [77]. The findings of both studies regarding the impact of PTMs on the function(s) of this checkpoint may contribute to the search for blocking antibodies targeting this checkpoint. In fact, one of the findings of these studies is the development of a monoclonal antibody, Ab-82, preferentially targeting B7H3 glycosylated at N91/309 and N104/322. According to the same authors, this antibody exhibited the ability to elicit cytotoxic T lymphocyte-mediated antitumor immunity via B7H3 internalization. It goes without saying that this particular finding needs to be supported by further investigations, preferably, clinical trials involving a high number of patients and controls. It is worth pointing out that currently, the most advanced B7H3 targeted drug in clinical trials is a B7H3 ADC, DS-7300, which successfully entered phase III clinical trials at the beginning of 2024 [80].

#### 2.3.2. B7-H3 as a Therapeutic Target

The overexpression of B7-H3 has been reported in a number of cancers, including non-small cell lung cancer (NSCLC) [81] and breast cancer [82]. The dual role of this protein in immune regulation and in disease progression has rendered it a highly attractive therapeutic target and a potential predictive biomarker in response to ICIs therapy. Earlier attempts to target this protein as a part of various therapeutic strategies have been met with little success. These attempts included various clinical trials in which monoclonal antibodies and bispecific antibodies were used to target B7-H3 [83,84]. More promising therapeutic results were obtained using antibody-drug conjugates (ADCs) to target this checkpoint. Basically, a conjugate combines a monoclonal antibody with cytotoxic agent, such combination exploits the specificity of the antibody and the capability of the agent to selectively deliver toxic drugs to target antigen-expressing cancer cells [85,86]. A number of ADCs targeting B7-H3 tested in various clinical trials have provided promising results; however, to date, there is no approved ADC to target this protein. Currently, there are many clinical trials assessing the efficacy of different ADCs in targeting B7-H3. These trials cover phases, I, II, and III. Presently, there is highly energetic efforts by various pharmaceutical and biotechnological companies, both big and small, to identify ADCs which can gain the approval of regulatory bodies. More information on these efforts can be found in recent publications [87,88,89,90,91,92]. Considering the current literature on B7-H3 as a therapeutic target, the following observations can be made: (i) As mentioned above, this protein can experience a number of PTMs, including glycosylation, which affects at least eight sites. Glycosylation of B7H3 can strongly influence its stability, localization, its role in cancer progression, resistance to therapy, and in immune evasion. The importance of this modification is not reflected in the results and conclusions of the majority of published clinical trials in which B7-H3 has been studied. The work in [73,77] is rather the exception. (ii) Various investigations have demonstrated that B7-H3 regulates cancer progression through multiple signaling pathways including, JAK2/STAT3, NF-Κb, PI3K/AKT, and ERK [93,94]. Many forms of cancer are often accompanied by an increase in M2 tumor-associated macrophages (TAMs), which can lead to poor prognosis of the investigated tumor. It has been suggested that TAMs can be pharmacologically suppressed by inhibiting the JAK2/STAT3 signaling pathway [94]. This observation was based on a study which investigated the use of a traditional Chinese medicinal formula to compact the progression of colorectal cancer (CRC) [94]. This study and others suggest that the suppression of certain pathways can be an important element in the search for a therapeutic strategy targeting B7-H3.

#### 2.3.3. B and T Lymphocyte Attenuator (BTLA)

**Figure 2:**
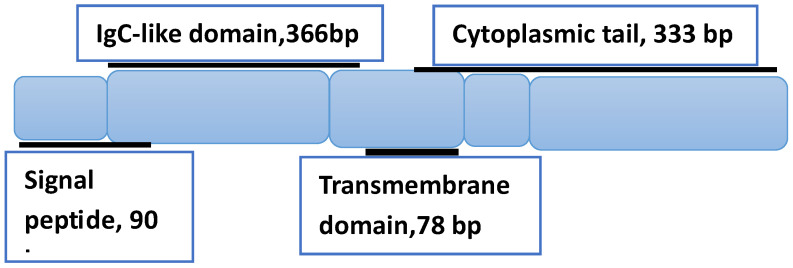
Schematic representation of BTLA’s structure, showing the three domains and associated number of base pairs (bp). The structure is based on Ref. [95].

BTLA is a co-inhibitory receptor of the B7 family that shares structural similarities with two extensively studied immune checkpoints, the cytotoxic T lymphocyte-associated antigen-4 (CTLA-4) and programmed death-1 (PD-1). This transmembrane glycoprotein has 289 amino acids and a molecular weight of 33 k Da. It consists of a signal peptide, an IgC-like extracellular domain, a transmembrane domain, and a cytoplasmic domain [95] (see Figure 2).

The interaction of this protein with the herpes virus entry mediator (HVEM) is known to negatively impact various immune responses. Such interaction is rather interesting, since it bridges two different protein families, the tumor necrosis factor receptor (TNFR) superfamily and the B7 family, which explains the role of this interaction in broad and important immune activities [95,96]. To assess the correlation between the BTLA expression level and the prognosis of EOC patients, a clinical trial involving 254 patients suffering different phases of epithelial ovarian carcinomas (EOC) was conducted [97]. Cancerous tissues taken from patients suffering different stages of the disease (I, II, III, IV) were examined by RT-PCR. This investigation concluded that the detection of BTLA in cancerous tissues can be considered an indication of a poor outcome in EOC patients. The same trial has also concluded that the inhibition of BTLA in combination with chemotherapy can enhance the immune reaction, resulting in strong antitumor effects. These conclusions are useful for the ongoing efforts to identify new prognostic biomarkers for the form of cancer being investigated. That said, this study has a number of limitations: (i) It is well established that BTLA shares a structural similarity with the PD-1 checkpoint. Both PD-1 and PD-L1 ligand are known to experience a number of PTMs, including extensive glycosylation [98,99]. This PTM has been demonstrated to negatively impact the reliability of PD-L1 as a predictive biomarker commonly used to identify patients who are likely to benefit from ICIs therapy. Little is known about the PTMs of BTLA, which may explain why such probable modifications were not considered in the interpretation of the acquired data reported in ref. [97]. (ii) What is the expression level of BTLA in the investigated tissues that represents a prognostic biomarker of the disease? The absence of an answer to this question by the above trial is due to the absence of controls, which would have provided information on the level of such protein in cancer-free tissues. To go beyond the determination of BTLA expression as the main prognostic parameter, a number of studies seek to gain more information on the biology of this checkpoint and its interaction with other proteins and associated signaling pathways. Protein–protein interactions are central to most biological processes. Such interactions within a given cell (also known as interactome) can give crucial information on checkpoint(s) complexation with other proteins and how such complexation influences the various signaling pathways associated with these checkpoints. The first large scale protein–protein interactions (interactome) were presented over 25 years ago [100,101]. A number of investigations have used this approach to examine the complexation and interaction of other proteins and the impact of such complexation on certain signaling pathways relevant to immunotherapy. Quantitative Interactomics was used to examine the rationale for concomitant PD-1 and BTLA co-inhibitor blockade in cancer immunotherapy [102]. The same study assessed the role of the SHP-1 and SHP-2 protein-tyrosine phosphatases in mediating PD-1 co-inhibition. According to this study, PD-1 predominantly recruits SHP-2, but when absent it recruits SHP-1 and remains functional. On the other hand, BTLA mainly recruits SHP-1 and to a lesser extent SHP-2. To appreciate the impact of these interactions on the BTLA signaling, a closer look at the structure of this protein is warranted. The cytoplasmic region of BTLA is known to consist of three highly conserved tyrosine-containing motifs: an immunoreceptor tyrosine inhibitory motif (ITIM), an immunoreceptor tyrosine-based switch motif (ITSM), and a growth-factor receptor-bound protein 2 (Grb2) binding site [103]. The presence of two inhibitory motifs (ITIM, ITSM), together with the stimulatory Grb-2 binding site renders BTLA a rare immune checkpoint receptor with possible bi-directional signaling [95]. These authors suggested that such bi-directional signaling starts with an interaction between BTLA and herpes virus entry mediator (HVEM), the resulting BTLA-HVEM complexation induces phosphorylation of both tyrosine residues within ITIMs and ITSMs. Such phosphorylation is necessary for the recruitment of tyrosine phosphatases SHP-1 and SHP-2. Earlier studies have shown that complexation between BTLA and SHP-1 prevents the phosphorylation of both CD28 and CD3ζ, resulting in the inhibition of T cell activation [104]. The other direction of signaling induced by Grb-2 protein binding to the Grb-2 motif, which recruits the PI3K kinase subunit p85, leads to activation of the PI3K/Akt signaling pathway.

#### 2.3.4. BTL Including Its Soluble Form as a Therapeutic Target

Due to alternative RNA splicing, this protein can be produced in a soluble form (sBTLA) [105]. The exact mechanism behind this process is still under investigation. Several works have attributed various roles to this soluble form. In solid tumors, high levels of sBTLA in sera have been associated with decreased survival in different cancers, including prostate, ovarian, and pancreatic adenocarcinoma [106,107,108]. BTLA’s role in immune regulation has made it a promising target for cancer immunotherapy, where blocking its inhibitory effects can restore T cell activity and enhance antitumor responses. Over the last few years, the BTLA/HVEM axis has emerged as a promising target for cancer immunotherapy. Part of these research efforts has focused on circulating BTLA, which has revealed to be a promising blood-based predictive biomarker of immunotherapy response in various cancers. Various investigations have revealed the role of the BTLA/HVEM axis in T cell dysfunction. Addressing T cell exhaustion is an important part of the research efforts to enforce the frontline defenders of the immune system. Development of antibodies capable of blocking BTLA is part of these efforts. A fairly limited number of patients (67) with advanced non-small cell lung cancer (NSCLC) or with refractory extensive-stage small cell lung cancer (SCLC) were enrolled for phase I/II of a clinical trial of Tifcemalimab (a recombinant humanized IgG4k monoclonal antibody targeting BTLA) combined with Toripalimab, a therapeutic monoclonal anti-PD-1 antibody with high binding affinity to PD-1 and enhanced potency to activate human T cells [109]. The trial was conducted to assess the safety and preliminary efficacy of tifcemalimab plus toripalimab in advanced lung cancer. The authors concluded that tifcemalimab plus toripalimab showed promising antitumor activities with acceptable safety, especially in advanced refractory SCLC.

In another clinical trial, patients (71) with relapsed or refractory lymphoma were enrolled in a 2-part, phase I study (NCT04477772) [110]. This phase I study was designed to assess the safety and preliminary efficacy of tifcemalimab (anti-BTLA monoclonal antibody) with or without toripalimab (anti-PD-1) in patients with relapsed or refractory lymphoma. This trial concluded that tifcemalimab as monotherapy or in combination with toripalimab demonstrated a favorable safety profile in lymphoma patients. Furthermore, tifcemalimab in combination with toripalimab showed promising clinical efficacy among patients with classical Hodgkin’s lymphoma who had previously received PD-(L)1 blockades.

Both trials cited here demonstrate that the choice of BTLA as a therapeutic target may contribute to current efforts to discover new classes of antibodies to combat certain forms of cancer. That said, the encouraging results reported in both trials are preliminary and involved a very limited number of patients, and therefore other trials, involving a much higher number of patients, and if possible different forms of cancer, are necessary before the enthusiasm for the initial positive results can be confirmed.

### 2.4. The Search for Predictive Biomarkers for IrAEs

**Figure 3:**
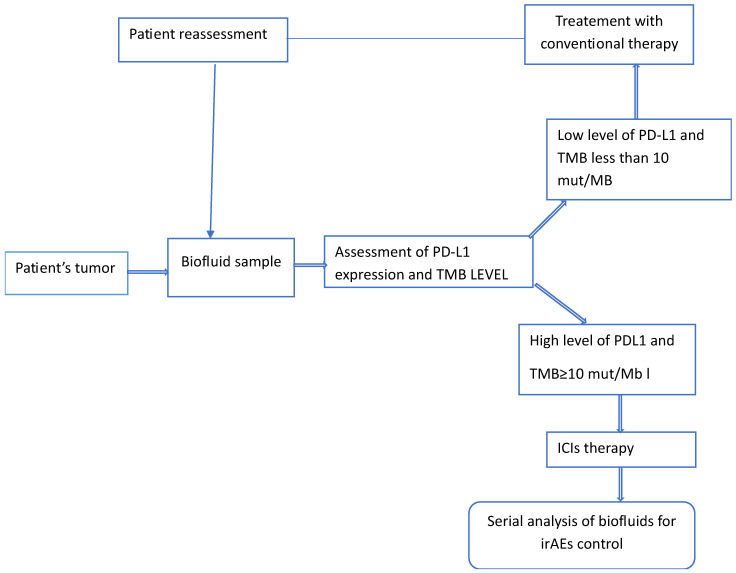
Schematic representation of the various steps a patient’s tumor is likely to go through when receiving ICIs therapy. The two predictive biomarkers, PD-L1 and MB, can be replaced by other biomarkers, depending on the type and on the stage of the disease.

The introduction of ICIs is still considered a major success in immunotherapy for the treatment of aggressive forms of cancer. To consolidate such success, further efforts have to be made to reduce or even avoid the potential severe irAEs associated with this therapy. These adverse events encompass a wide range of toxicities, including gastrointestinal, cardiovascular, and nervous system toxicities (see Table 1 and Figure 3).

Given the wide range of clinical consequences of these adverse events, early diagnosis and timely management of these events are crucial in minimizing the risks of serious reactions to immunotherapy. The increasing use of ICIs in the treatment of various cancers, and the high percentage of the same patients developing irAEs, enhanced research efforts for the discovery of effective biomarkers for the early detection of these adverse events. The current literature reports different experimental approaches in the search for effective biomarkers for a number of these adverse events. The use of serial liquid biopsy (LB) in the analysis of blood samples of cancer patients is one of the methods available to gain reliable information on these biomarkers [111,112]. This sampling method was used to investigate two adverse effects, early death (ED) and hyper-progression (HPD), two effects associated with the ICIs treatment of advanced non-small cell lung cancer (aNSCLC) patients. Plasma samples were collected at baseline (T1) and after 3–4 weeks of treatment (T2). According to this investigation, both effects were observed in aNSCLC patients expressing high levels of PD-L1. In the absence of specific biomarkers for the two adverse effects and the lack of a clinical criteria for their investigation, the authors adapted what they called potential clinical criteria. Basically, patients experiencing death within 12 weeks of starting ICIs therapy represented the basis of such criteria. Cell-free DNA (cfDNA) quantification and variant allele fraction (VAF) of tumor-associated genetic alterations were assessed to establish the correlation between these parameters and the investigated side effects. This study reported that in the case of ED, extensive variation in cfDNA concentration between T1 and T2 was observed, but there was no significant change in the highest VAF within the same time window. No detectable variation in cfDNA was observed for HPD patients, while variation in maxVAF contributed to the identification of patients experiencing HPD.

Currently, there are insufficient real world clinical data to allow accurate categorization of reliable biomarkers for irAEs prediction and/or diagnosis. That said, a recent article categorized such potential biomarkers into blood cell-based, cytokine/chemokine, immunoglobulin/other secreted proteins, and immunogenetic biomarkers [16]. Assessment of cytokine/chemokine in biofluids of cancer patients is frequently performed in the search for reliable biomarkers for the early detection of irAEs. Chemokines are small proteins with a molecular weight in the range of 8–15 kD. Based on the number and arrangement of conserved L-Cysteine at the N-terminal, this family of proteins is classified into four subfamilies, C, CC, CXC, and CX3C. These signaling proteins are secreted by various cells and are known to play key roles in inducing chemotaxis, promoting differentiation and multiplication of leukocytes, and causing tissue extravasation [113]. In one of these studies, the levels of 40 cytokines were determined in a number of cancer patients receiving ICIs therapy, and these levels were compared with those taken from a smaller number of controls. Cytokine levels were measured in sera samples prior to treatment, after 2–3 weeks, and after 6 weeks and analyzed for correlation with the development of irAEs [114]. This study concluded that patients who developed irAEs had lower baseline levels and greater post-treatment increases in multiple cytokines. Correlation between cytokine changes and irAEs were examined for 52 melanoma patients receiving ICIs therapy. Serum samples provided by these patients were examined using Luminex serum assay [115]. The measurements were performed at baseline, 1, 2, and 3 months after starting ICIs. In this retrospective study, the authors assessed the longitudinal expression of 34 cytokines in patients who received anti-PD-1, anti-CTLA-4, or combined anti-PD-1 and anti-CTLA-4. Despite a low number of patients, the same study confirmed a number of earlier observations regarding the correlation between some cytokine levels and certain irAEs. The authors observed that more than half of the tested patients who developed grade 1–2 irAEs showed no change in baseline cytokine levels. Eight patients who developed dermatitis showed elevated plasma concentration of ANG-1 and CD40L compared to patients who did not develop the disease, while four patients who developed pneumonitis showed elevated baseline plasma concentration of IL-17 and decreased baseline concentration of IL-8. Among eight patients who developed colitis, there was a trend toward decreased baseline concentration of cytokine GCSF. Previous studies have shown that the two cytokines ANG-1 and IL-17 are of therapeutic interest. The Ang/Tie2 axis and its signal transduction are known to play an important role in the regulation of vascular stability, angiogenesis and inflammation. Targeting the Ang/Tie2 signal axis can normalize micro vessels in psoriatic lesions [116]. Since its discovery nearly 30 years ago, IL-17 has emerged as a key cytokine for host protection against mucosal infections but also as a major pathogenic cytokine and drug target in multiple autoimmune and inflammatory diseases [117,118].

## 3. Conclusions and Future Perspectives

The unprecedented success of immune checkpoint inhibitors in the treatment of a number of advanced forms of cancer has been undermined by two limitations: the first is the low number of patients who responded to the therapy, and the second is the development of serious adverse events as a result of such therapy. The last decade has witnessed intense research activities to discover and develop predictive biomarkers to identify the likely responders to the therapy, and to detect early-stage adverse events associated with ICIs therapy. These activities included numerous clinical trials. In this text we discussed both well established as well as potential biomarkers undergoing clinical assessment. Based on such discussion and considering expert opinions cited in this text, the following considerations can be made: (i) None of the three predictive biomarkers approved by the FDA can give an accurate determination of the response to ICIs therapy. The combination of two markers improves the outcome but remains below expectation. The predictive efficacy of PD-L1 and TMB is still strongly debated. The first is based on measuring its expression level on cancerous cells, while the second biomarker is based on measuring the total number of somatic non-synonymous mutations present within a cancer genome. Two approved biomarkers are based on two different mechanisms, yet contradictory results in performance are common in both cases. This observation may suggest that the inconsistent performance reported by different studies is not an intrinsic characteristic of either biomarker. Certainly, the biological and the chemical characteristics of the detected entity have a role to play; however, a bigger role in this inconsistent performance can be attributed to the detection assay and the method of measurement used. As mentioned in this text, TMB can be accurately measured by using WGS; however, the high costs and relatively long times of implementation rendered it a rare clinical commodity. Instead, a less accurate method of multiple commercial gene panels is commonly used clinically for TMB estimation. Future predictive biomarkers for irAEs currently under assessment are likely to face similar problems of achieving consistent efficacy, unless the question of implementation is tightly monitored. It is fair to say that the performance of existing predictive biomarkers, either approved or still under investigation, needs to be re-evaluated through well designed clinical trials, involving the highest numbers of patients and controls. These trials should employ cutting edge genetic and proteomic-based technologies, ensuring rigid standardization and strict coordination between the various clinics and research groups organizing such trials. The increasing use of spatial omics technologies with subcellular level resolution is a highly promising approach and is likely to open new possibilities in the area of spatial biomarkers.

In conclusion, there is an important aspect which is not given sufficient attention in the search for potential predictive biomarkers. This observation is based on the following consideration. Several potential biomarkers are proteins. These proteins will experience PTMs, interact with other proteins, and have specific signaling pathways. The current literature on the discovery and development of predictive biomarkers demonstrates a paucity of studies in which the three parameters are considered contemporarily in the investigation of a given potential protein biomarker.

## Figures and Tables

**Table 1:** A sample of irAEs, which can be provoked by ICIs therapy.

Some irAEs Associated with ICIs Therapy	Observations	Ref.
Hepatotoxicity	This toxicity has a variable profile of both symptoms and severity, which renders it difficult to diagnose. It has a relatively low rate (1–2% in the case of anti-PD-1therapy) and a much higher rate (13–16% associated with combined CTLA-4/PD-1).	[1]
Endocrine toxicity	This adverse event primarily affects the thyroid gland; it is more common in anti-PD-1 therapy. In lung cancer treatment, the incidence of this event was about 18%.	[2]
Skin toxicity	Skin toxicity is very common in patients treated with ICIs. Incidence of 90% for patients treated with CTLA-4 and 70% for those treated with PD-1/PD-L1.	[3]
Neurotoxicity	Neurotoxicity associated with ICIs therapy is rather rare, yet it can result in a high rate of fatality. Due to their clinical diversity, diagnosing these forms of neurotoxicity remains a challenging task.	[4]
Cardiac toxicity	Cardiac toxicity due to ICIs treatment is rather rare (1–2%), yet because of its high potential lethality it has to be under close and continuous monitoring.	[5]
Lung toxicity	Pulmonary toxicity occurs in less than 3% of the treated patients. Patients treated with PD-1 inhibitor were more exposed to this toxicity compared to those treated with PD-L1.	[6]
Dermatologic toxicities	Cutaneous toxicities are the most common irAEs, which can affect 60–70% of patients treated with a combination of anti-PD-1/PD-L1 and anti-CTLA-4 inhibitors.	[7]
Renal toxicities	Acute kidney injury is closely associated with ICIs therapy, occurs in about 5% of patients receiving a combination of ICI therapy, and 2% of those treated with ICI monotherapy.	[8]

**Table 2:** Predictive biomarkers in response to ICIs therapy. The top three biomarkers have been already approved by the FDA, while the other two (B7-H3 and BTLA) are still under investigation as potential predictive biomarkers.

Predictive Biomarkers for ICIs Response	Observations
Programmed Death Ligand 1 (PD-L1)	PD-L1 was approved by the FDA in2015 as a predictive biomarker in response to ICIs treatment of non-small cell lung cancer (NSCLC). Different assays-approved by the FDA use immunohistochemistry (IHC). The use of different assays and different modes of implementation are considered responsible for inconsistency in reported results.
Tumor Mutational Burden (TMB).	TMB was approved by the FDA in 2020 as a predictive biomarker in response to ICIs treatment of unresectable or metastatic solid tumors. This biomarker measures the total number of somatic non-synonymous mutations present within a cancer genome. Reported inconsistencies in clinical trials are commonly attributed to infrequent use of whole-genome sequencing (WGS) in clinical practice.
Microsatellite Instability/Defective Mismatch Repair (MSI/dMMR).	MSI/dMMR was the second predictive biomarker to be approved by the FDA in 2017. This marker is designated for measuring response to ICIs treatment against unresectable or metastatic solid tumors. There are three different assays available in clinical tests: IHC for detecting dMMR, PCR, and NGS for detecting MSI. As is the case with PD-L1, inconsistency in the reported results is due to the use of different assays and different approaches in their implementation.
Homolog 3 protein (B7-H3), also known as CD276.	High expression level of this protein compared to healthy tissues is the main parameter under investigation to establish whether such differences can be considered a predictive biomarker for advanced solid tumors.
B and T lymphocyte attenuator (BTLA).	This co-inhibitory receptor shares structural similarity with two extensively studied immune checkpoints, 4 CTLA-4 and PD-1. The correlation between BTLA expression and the prognosis of different forms of cancer is under investigation.

**Table 3:** A list of immune checkpoints, both well researched and emerging, which are the subjects of clinical and pharmaceutical investigations for the development of new immune therapeutic strategies and/or the discovery of predictive biomarkers in response to ICIs and associated irAEs.

Immune Checkpoints
Cell death ligand-L1(PD-L1) Programmed cell death-1 (PD-1) Cytotoxic T lymphocyte antigen-4 (CTLA-4) Lymphocyte activation gene-3 (LAG-3) T cell immunoglobulin and ITIM domain (TIGIT) T cell immunoglobulin and mucin-domain containing-3 (TIM-3) V-domain immunoglobulin suppressor of T cell activation (VISTA) B7 homolog 3 protein (B7-H3) Inducible T cell costimulatory (ICOS) B and T lymphocyte attenuator (BTLA)

## Data Availability

No new data were created or analyzed in this study. Data sharing is not applicable to this article.

## Abbreviations

ICIs	Immune checkpoint inhibitors.
IrAES	Immune-related adverse events.
CTLA-4	Cytotoxic T lymphocyte-associated antigen 4.
PD-1	Programmed death cell protein.
PD-L1	Programmed death cell protein legend-1.
MSI/Dmmr	Microsatellite instability/defective mismatch repair.
TMB	Tumor mutational burden.
LAG-3	Lymphocyte activation gene-3.
TIGIT	T cell immunoglobulin and ITIM domain.
TIM-3	T cell immunoglobulin and mucin-domain containing-3.
VISTA	V-domain immunoglobulin suppressor of T cell activation.
B7-H3	B7 homolog 3 protein.
ICOS	Inducible T cell costimulatory.
BTLA	BTLA B and T lymphocyte attenuator.
PTMs	Post-translational modifications.
LC-MS/MS	Liquid chromatography/tandem mass spectrometry.
MALDI-TOF-MS	Matrix-assisted laser desorption ionization-mass spectrometry.
aNSCLC	Advanced non-small cell lung cancer.
rwOS	Real world overall survival.
IgV	Immunoglobulin variable-like domain.
IgC	Immunoglobulin constant-like domain.
NK	Natural killer cells.
qRT-PCR	Quantitative real-time polymerase chain reaction.
WB	Western blotting.
ELISA	Enzyme-linked immunosorbent assay.
HVEM	Herpes virus entry mediator.
EOC	Epithelial ovarian carcinomas.
ITIM	Immunoreceptor tyrosine inhibitory motif.
ITSM	ITSM Immunoreceptor tyrosine-based switch motif.
Grb2	Growth-factor receptor-bound protein.
cfDNA	Cell-free DNA.
